# Comprehensive analysis of the microbiome and metabolome in pus from pyogenic liver abscess patients with and without diabetes mellitus

**DOI:** 10.3389/fmicb.2023.1211835

**Published:** 2023-06-23

**Authors:** Yawen Guo, Hairui Wang, Zhaoyu Liu, Zhihui Chang

**Affiliations:** Department of Radiology, Shengjing Hospital of China Medical University, Shenyang, China

**Keywords:** bacterial composition, pyogenic liver abscess, 16S rDNA sequencing, untargeted metabolomics analysis, metabolome

## Abstract

**Introduction:**

Pyogenic liver abscess (PLA) patients combined with diabetes mellitus (DM) tend to have more severe clinical manifestations than without DM. The mechanism responsible for this phenomenon is not entirely clear. The current study therefore aimed to comprehensively analyze the microbiome composition and metabolome in pus from PLA patients with and without DM, to determine the potential reasons for these differences.

**Methods:**

Clinical data from 290 PLA patients were collected retrospectively. We analyzed the pus microbiota using 16S rDNA sequencing in 62 PLA patients. In addition, the pus metabolomes of 38 pus samples were characterized by untargeted metabolomics analysis. Correlation analyses of microbiota, metabolites and laboratory findings were performed to identify significant associations.

**Results:**

PLA patients with DM had more severe clinical manifestations than PLA patients without DM. There were 17 discriminating genera between the two groups at the genus level, among which *Klebsiella* was the most discriminating taxa. The ABC transporters was the most significant differential metabolic pathway predicted by PICRUSt2. Untargeted metabolomics analysis showed that concentrations of various metabolites were significantly different between the two groups and seven metabolites were enriched in the ABC transporters pathway. Phosphoric acid, taurine, and orthophosphate in the ABC transporters pathway were negatively correlated with the relative abundance of *Klebsiella* and the blood glucose level.

**Discussion:**

The results showed that the relative abundance of *Klebsiella* in the pus cavity of PLA patients with DM was higher than those without DM, accompanied by changes of various metabolites and metabolic pathways, which may be associated with more severe clinical manifestations.

## Introduction

Pyogenic liver abscess (PLA) is a common intraperitoneal infection with several risk factors, such as diabetes mellitus (DM) and underlying hepatobiliary or pancreatic disease (Chadwick et al., 2018; Mukthinuthalapati et al., 2020). Although the mortality rate has improved, a significant percentage of PLA patients develop complications including extrahepatic migratory infection (EMI), abscess rupture and so on (Mavilia et al., 2016). DM is a predisposing factor for PLA (Thomsen et al., 2007; Mavilia et al., 2016); PLA patients with DM tend to have a higher prevalence of metastatic infection, bacteremia, multi-organ dysfunction syndrome and intensive care unit (ICU) admission (Foo et al., 2010; Tian et al., 2012). The mechanism underlying this phenomenon is not fully understood, so it is important to better understand the relevant mechanism, which can be used to develop preventive and treatment strategies.

Pathogenic microorganisms and their virulence levels are important factors affecting the prognosis of PLA (Liu et al., 2018). The gut microbiota play an important role in maintaining intestinal homeostasis and inducing the development of the intestinal immune system (Bouskra et al., 2008; Sonnenberg and Artis, 2012). Intestinal microorganisms entering the liver through the portal vein is one of the main causes of PLA (Lardière-Deguelte et al., 2015). Studies have reported a significant association between changes in the composition profile of gut microbiota and the development of DM (Gurung et al., 2020; Iatcu et al., 2021). In addition, studies have focused on the associations between metabolites and diabetes (Tang et al., 2019; Qi et al., 2022). Therefore, we speculated that the microbiome composition and metabolites in the pus cavity of PLA patients with DM were different from those of PLA patients without DM. A combination of 16SrDNA sequencing used for pathogen identification and untargeted metabolomics analyses for metabolite identification provided technical support to verify our hypothesis (Moon et al., 2021; Yu et al., 2021).

## Materials and methods

### Study population

The Ethics Committee of Shengjing Hospital of China Medical University approved this study (2022PS1067K) and written informed consent was obtained from all patients involved in the study for the publication of any potentially identifiable data included in this article. We retrieved the records of patients with a diagnosis of PLA from January 2017 to December 2021 from the electronic medical database at our institution (*n* = 290). According to the presence or absence of DM, these patients were divided into DM group (*n* = 189) and Non-DM group (*n* = 101).

Demographic information, laboratory findings, underlying diseases, hospital length of stay (LOS), the incidence of intensive care unit (ICU) admission and extrahepatic migratory infection (EMI) were obtained from medical records. Diagnostic criteria for PLA, DM, EMI, biliary tract disease and digestive system cancer were as previously described (Chang et al., 2015; Ren et al., 2020; Wang et al., 2022).

### Pus sample collection

**Figure 1:**
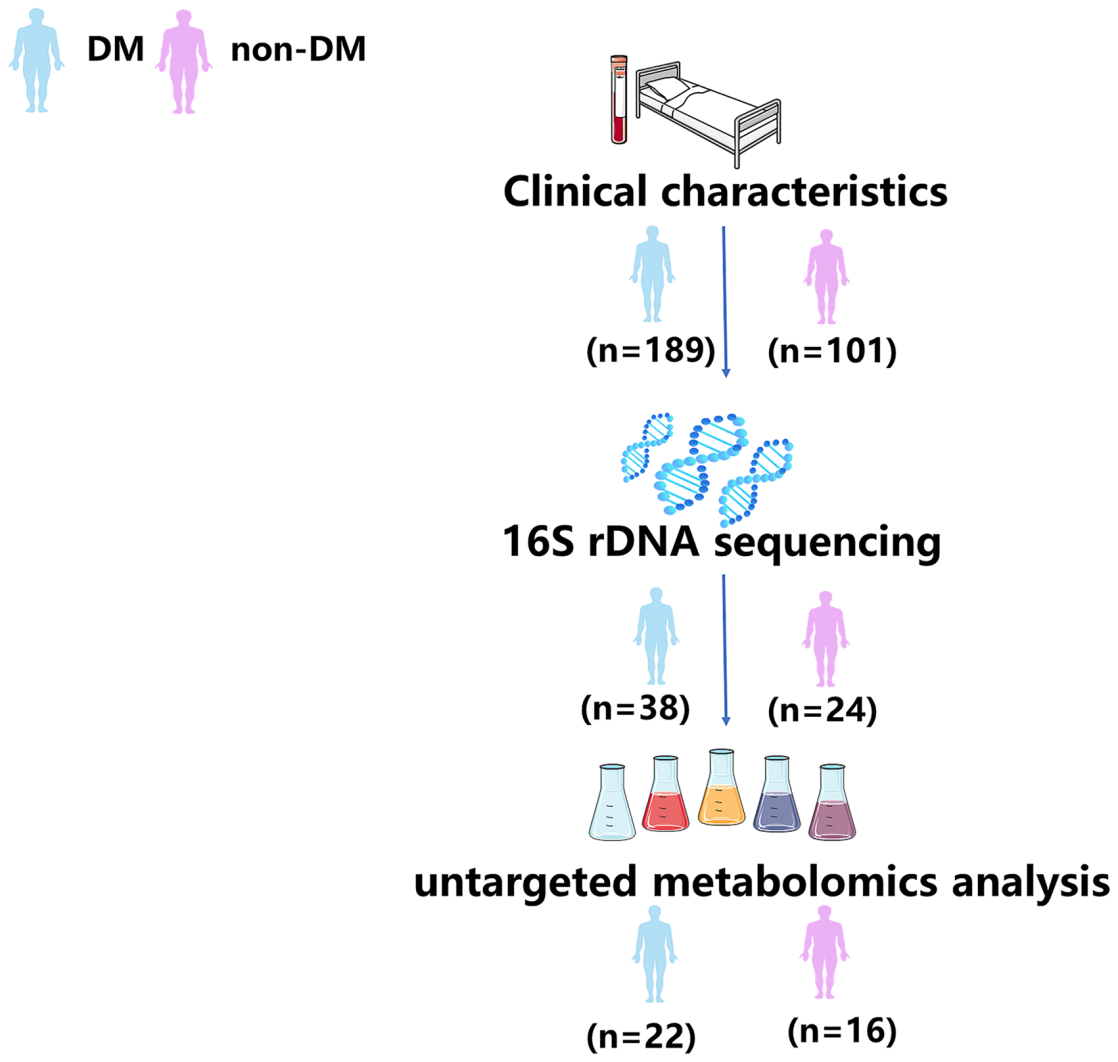
Workflow of the analysis.

From January 2020 to December 2021, a total of 62 pus samples were prospective and continuously collected for conventional culture and 16S rDNA sequencing. According to the presence or absence of DM, these samples were divided into DM group (*n* = 38) and Non-DM group (*n* = 24). Of these, 38 pus samples (DM group, *n* = 22; Non-DM group, *n* = 16) were collected for untargeted metabolomics analysis. All collected pus samples were analyzed and no samples were artificially removed. The workflow of this study is shown in Figure 1.

The inclusion criteria were: (a) presence of a focal lesion or lesions in the liver on contrast enhanced CT images; (b) frank pus aspirated from the abscess cavity through diagnostic, and/or surgical drainage procedures; and (c) positive microbiological culture results from liver abscess and/or blood cultures.

The methods used for abscess drainage and pus collection were as described in a previous study (Song et al., 2014). All percutaneous procedures which strictly follows the principle of asepsis were performed under ultrasound guidance. Eighteen gage P.T.C Chiba needles of varying lengths were used to puncture the abscesses. The aspirated isolates including cultures were sent for microbiological analysis, 16S rDNA gene sequencing and untargeted metabolomics analysis. Under fluoroscopic guidance, 2–4 mL of undiluted contrast media was instilled into the abscess cavity through the 18G needle and then a 0.035-inch wire was inserted into the abscess cavity. After serial dilatation, an 8.5F pigtail catheter was inserted into the abscess. After collection, pus samples were stored at −80°C in a biological sample bank until used for 16S rDNA sequencing and untargeted metabolomics analysis.

### 16S rDNA sequencing

The full details regarding microbiome methodology are provided in the Supporting Information. The 16S rDNA sequencing was performed to characterize the pus microbiome composition. Length-heterogeneity PCR fingerprinting was routinely used to rapidly survey our samples and standardize the community amplification. We then characterized the microbial taxa associated with the pus microbiome using Multitag Sequencing of samples. Statistical data analysis was performed based on the feature table and feature sequence obtained as described in the Supporting Information. The full analysis script is available in the Supporting Information. Alpha and beta diversity analyses were calculated using QIIME2 and all the graphs were constructed using the R package (R Foundation for Statistical Computing, Vienna, Austria). PICRUSt was used for inferred metagenomic analyses as previously described (Puri et al., 2018). Significant difference analysis was performed to compare the differential species at the genus level between the two groups (*p* < 0.05). Enrichment analysis of abundant taxa or functions between the two groups was performed using the linear discriminant analysis effect size (LEfSe) with a threshold logarithmic linear discriminant analysis (LDA) score set at 3.0. BugBase was used to predict the bacterial phenotype (Meyer et al., 2008; Liao et al., 2022).

### Untargeted metabolomics analysis

The full details regarding untargeted metabolomics analysis are provided in the Supporting Information. Here, we briefly describe the sample analysis. Partial Least-Squares Discriminant Analysis (PLS-DA) was conducted using metaX to discriminate the different variables between groups and the variable importance for the projection (VIP) value was calculated. A VIP cut-off value of 1.0 was used to select important features. A volcano plot was used to compare differential metabolites between the two groups. Kyoto Encyclopedia of Genes and Genomes (KEGG) enrichment analysis was performed on significantly different metabolites (satisfying ratio ≥ 2 or ratio ≤ 0.5; a value of *p* ≤ 0.05; VIP > 1). All graphs were constructed using the R package (R Foundation for Statistical Computing).

### Statistical analyses

Continuous variables were expressed as the mean ± standard deviation or median (interquartile range). Categorical variables were expressed as absolute numbers with frequencies (%). Differences between continuous data were analyzed using Student’s *t*-test or the Mann–Whitney U-test. Differences between categorical data were analyzed using the chi-squared test or Fisher’s exact test, as appropriate. All statistical analyses were performed using SPSS statistical software for Windows, version 25.0 (IBM, Armonk, NY, United States). Two-tailed *p*-values < 0.05 were considered statistically significant. Correlation heatmaps with signs were performed using the OmicStudio tools. Scatter plots were constructed using Prism 8 software (GraphPad, San Diego, CA, United States).

## Results

### Clinical characteristics of participants

A total of 290 PLA patients (DM, *n* = 189; non-DM, *n* = 101) were studied (Table 1). As expected, there are a number of laboratory findings of PLA patients in both DM and non-DM groups falling outside the reference range. For example, leukocyte, neutrophils (NEUT),NEUT% and so on. Compared with the non-DM group, PLA patients with DM had a higher C-reactive protein (CRP, an inflammatory marker)level, higher EMI incidence and longer LOS, which showed that PLA patients with DM have more severe clinical manifestations.

**Table 1:** Clinical characteristics of 290 pyogenic liver abscess patients.

Characteristic	Reference ranges	DM *n* = 189 (%)	Non-DM *n* = 101 (%)	*p*-value
Age (year)	–	57.57 ± 13.06	58.29 ± 14.53	0.603
**Laboratory findings**				
Leukocyte (10^9^/L)	3.5–9.5	11.90 ± 5.59	12.43 ± 4.78	0.281
NEUT (10^9^/L)	1.9–7.2	10.17 ± 6.34	10.20 ± 4.57	0.521
LY (10^9^/L)	1.1–2.7	1.35 ± 2.65	1.29 ± 0.67	0.186
NEUT (%)	42.3–71.5	95.83 ± 198.68	80.66 ± 8.85	0.359
LY (%)	16.8–43.4	21.42 ± 139.12	11.43 ± 6.55	0.600
PLT (10^9^/L)	135–350	246.79 ± 145.28	225.09 ± 123.22	0.248
PT (s)	9.4–12.5	14.25 ± 3.91	13.89 ± 2.86	0.086
INR	0.8–1.2	1.27 ± 0.34	1.63 ± 4.04	0.172
DD (μg/L)	0–252	2727.20 ± 8840.82	1961.56 ± 3805.93	0.511
ALB (g/L)	35–53	30.30 ± 6.16	29.95 ± 4.83	0.878
ALT (U/L)	0–40	83.92 ± 133.13	61.84 ± 53.58	0.250
AST (U/L)	5–34	69.88 ± 118.22	47.45 ± 46.94	0.434
TBIL (μmol/L)	3.4–20.5	16.10 ± 12.66	23.38 ± 41.00	0.159
CRP (mg/L)	0–6	178.70 ± 105.86	149.24 ± 89.22	**0.041**
Hospital LOS (days)	–	13.71 ± 8.73	12.93 ± 12.06	**0.026**
EMI	–	42 (22.22)	10 (9.90)	**0.009**
ICU admission	–	8 (4.23)	3 (2.97)	0.753
Death	–	3 (1.59)	1 (0.99)	1.000

NEUT, neutrophils; LY, lymphocytes; PLT, platelets; PT, prothrombin time; INR, international standardized ratio; DD, D-dimer; ALB, albumin; ALT, alanine aminotransferase; AST, aspartate transaminase; TBIL, total bilirubin; CRP, C-reactive protein; LOS, length of stay; EMI, extrahepatic migratory infection; ICU, intensive care unit. Bold value indicates *p* value <0.05.

### Pus microbiota analysis

To determine the effects of DM on the pus microbiome composition in PLA patients, pus samples of 62 consecutive PLA patients (DM, *n* = 38; non-DM, n = 24) were performed using 16S rDNA sequencing. The clinical data of these 62 patients are shown in Table 2. As expected, compared with non-DM patients, PLA patients with DM had a higher CRP level. Although there was a statistical difference in platelets (PLT) in Table 2, the PLT in both groups were all within the reference range and had no clinical significance. Besides, there was no statistical difference in PLT in Table 1. Therefore, we speculate that the statistical difference in PLT in Table 2 may be due to the small sample size.

**Table 2:** Clinical characteristics of 62 pyogenic liver abscess patients.

Characteristic	Reference ranges	DM *n* = 38 (%)	Non-DM *n* = 24 (%)	*p*-value
Age (year)	–	58.84 ± 12.43	53.00 ± 13.99	0.087
**Underlying diseases**				
Biliary tract disease	–	2 (5.26%)	7 (29.17%)	**0.021**
Digestive system cancer	–	2 (5.26%)	4 (16.67%)	0.195
**Laboratory findings**				
Leukocyte (10^9^/L)	3.5–9.5	12.04 ± 5.04	11.91 ± 4.13	0.983
NEUT (10^9^/L)	1.9–7.2	10.04 ± 4.85	9.97 ± 3.84	0.851
LY (10^9^/L)	1.1–2.7	1.06 ± 0.68	1.05 ± 0.53	0.868
NEUT%	42.3–71.5	82.36 ± 8.64	82.72 ± 7.90	0.937
LY%	16.8–43.4	9.53 ± 6.26	9.33 ± 4.77	0.729
PLT (10^9^/L)	135–350	276.74 ± 133.96	214.63 ± 122.42	**0.040**
PT (s)	9.4–12.5	15.27 ± 7.72	14.23 ± 2.76	0.743
INR	0.8–1.2	1.38 ± 0.65	1.30 ± 0.25	0.708
DD (μg/L)	0–252	2606.66 ± 5459.11	1854.30 ± 2054.64	0.935
ALB (g/L)	35–53	29.10 ± 4.78	31.41 ± 5.99	0.082
ALT (U/L)	0–40	66.38 ± 73.53	69.08 ± 58.93	0.784
AST (U/L)	5–34	55.84 ± 94.11	50.42 ± 48.17	0.879
TBIL (μmol/L)	3.4–20.5	16.76 ± 11.99	19.67 ± 16.50	0.608
CRP (mg/L)	0–6	183.07 ± 83.09	135.12 ± 61.58	**0.033**
ALBI	–	−1.72 ± 0.44	−1.89 ± 0.53	0.272
Hospital LOS (days)	–	12.61 ± 8.31	9.42 ± 4.72	0.165
EMI	–	3 (7.89%)	2 (8.33%)	1.000

The score of ALBI = (log_10_ TBIL × 0.66) + (−0.085 × ALB).

NEUT, neutrophils; LY, lymphocytes; PLT, platelets; PT, prothrombin time; INR, international standardized ratio; DD, D-dimer; ALB, albumin; ALT, alanine aminotransferase; AST, aspartate transaminase; TBIL, total bilirubin; CRP, C-reactive protein; ALBI, albumin-bilirubin; EMI, extrahepatic migratory infection. Bold value indicates *p* value <0.05.

Supplementary Table S1 shows that 16SrDNA sequencing was able to detect the microbiome composition in conventionally cultured negative samples and was able to detect more bacteria in samples that were conventionally cultured as a single strain.

### Alpha and beta diversities of the pus microbiota

**Figure 2:**
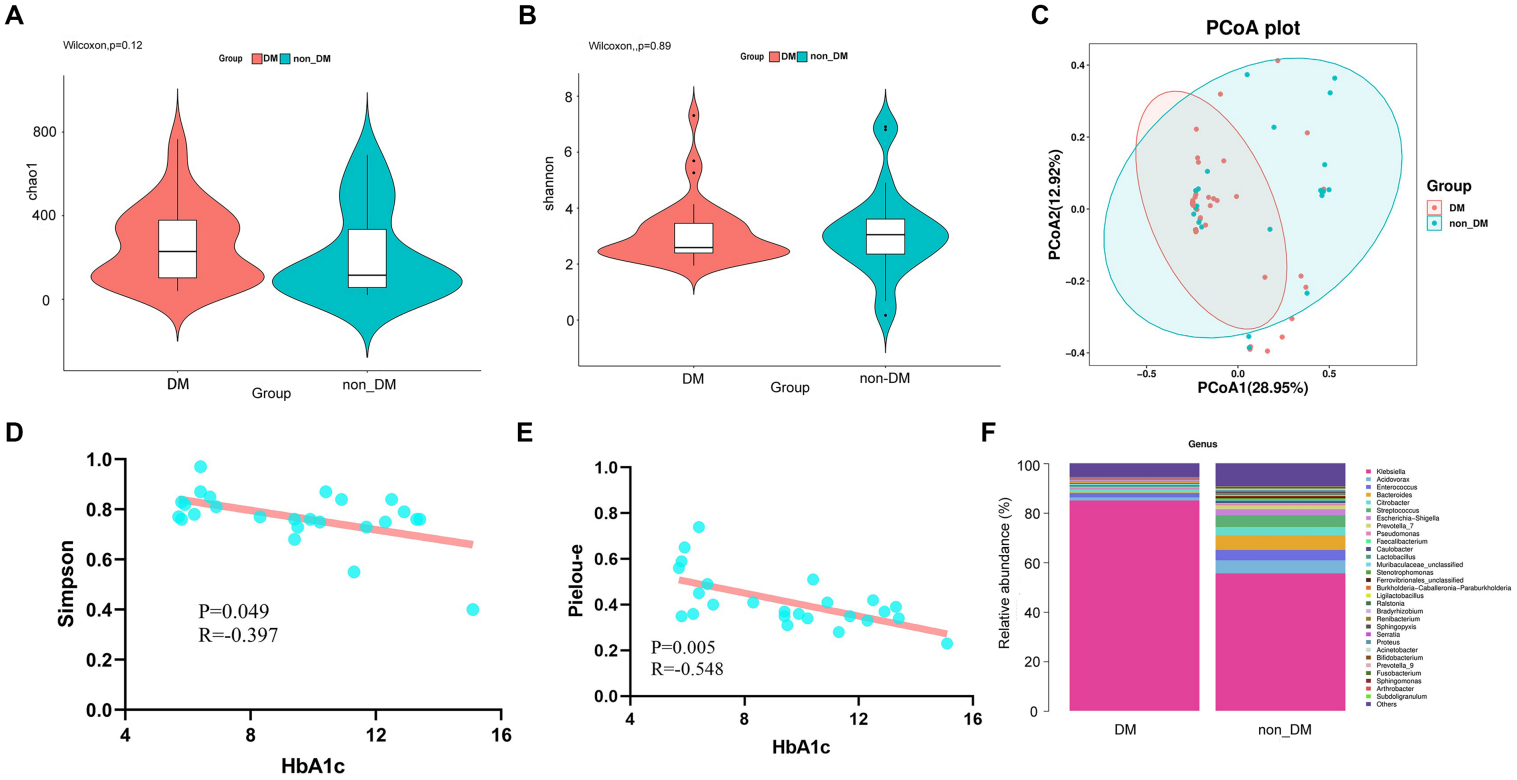
Alpha diversity, beta diversity, and taxonomy community of 16SrDNA sequencing and linear correlation analysis. **(A)** Chao 1 index of the diabetes mellitus (DM) and non-DM groups. **(B)** Shannon diversity index of the DM and non-DM groups. **(C)** Beta diversity analyzed by Bray Curtis PCoA. **(D)** Scatter plot of the correlation between Simpson diversity index and HbA1c. **(E)** Scatter plot of the correlation between the Pielou-e diversity index and HbA1c. **(F)** Taxonomic percentages according to compositions at the genus level.

Alpha diversity represents the species evenness and richness within the microbiota, while beta diversity represents the shared diversity within the microbiota at different ecological distances (Yang et al., 2022). The Chao 1 index describing species richness revealed no significant difference (DM vs. non-DM; *p* = 0.12) (Figure 2A). In addition, the Shannon diversity index did not show significant difference between the DM vs. non-DM patients; *p* = 0.89 (Figure 2B). We then evaluated the beta diversity of the two groups. Using Bray Curtis PCoA, we found some differences in species classifications between DM and non-DM patients (Figure 2C), but there was no statistical difference. We then conducted a correlation analysis between Chao1, Simpson, Shannon and Pielou-e indices of α diversity and clinical data, which suggested (Supplementary Table S2) that the Simpson and Pielou-e indices were negatively correlated with glycosylated hemoglobin HbA1c levels (Figures 2D,E).

### Alterations in pus microbiome composition due to DM

To determine the effects of DM, we first compared the microbial taxa in DM vs non-DM patients. Compared with non-DM patients, at the genus level, DM patients showed significantly greater relative abundance of *Klebsiella* (Figure 2F). Additionally, DM patients showed lesser levels of the genera, *Acidovorax*, *Enterococcus*, *Bacteroides*, *Citrobacter* and *Streptococcus*, when compared with the non-DM group.

### Microbiome signatures for PLA patients with DM were distinct from non-DM patients

**Figure 3:**
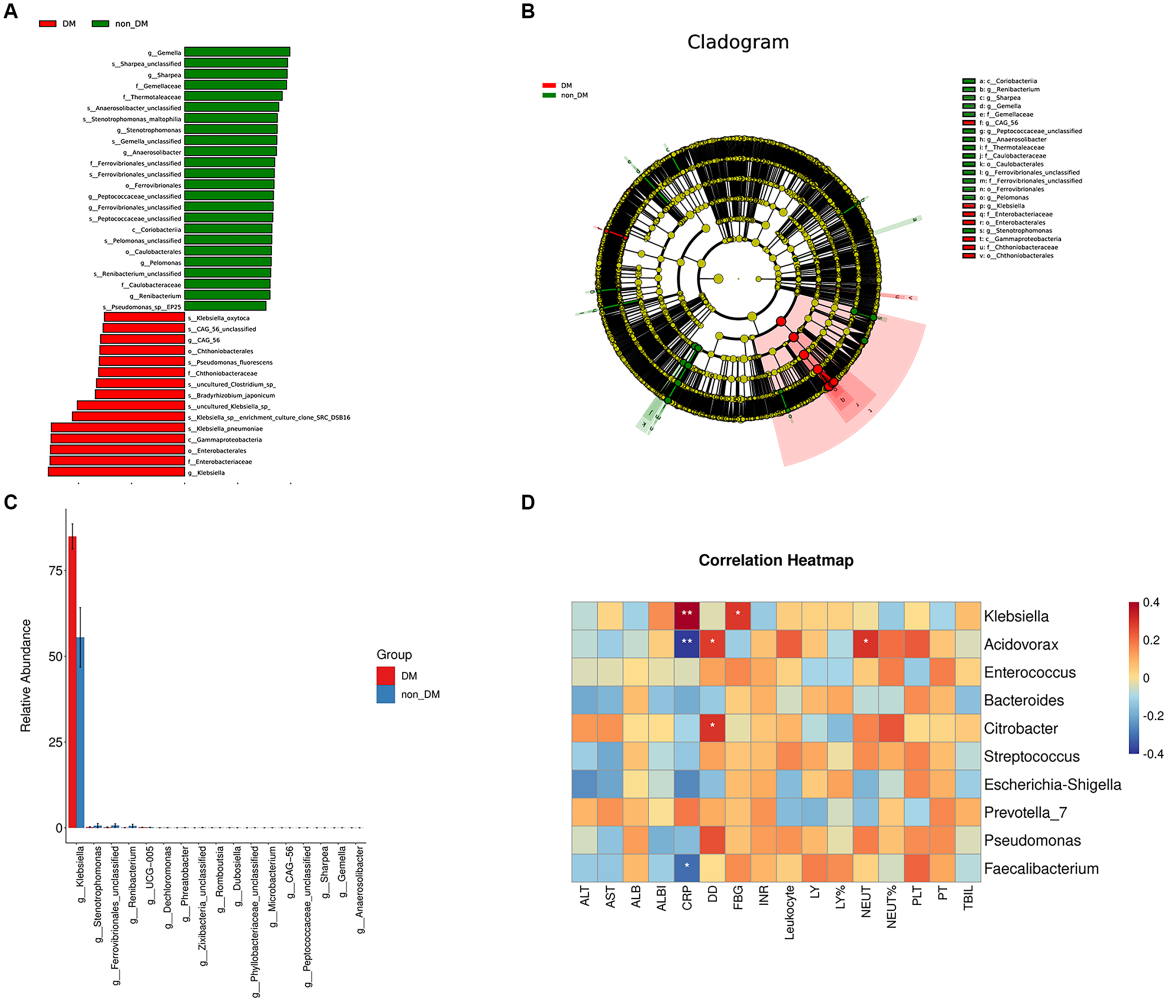
LEfSe analysis, microbial difference analysis, and heatmap of correlation analysis. **(A)** Histogram of the logarithmic linear discriminant analysis (LD) value distribution (LDA score > 3.0; *p* < 0.05). A longer bar size corresponds to a larger enrichment of the group. **(B)** Cladogram. **(C)** Analysis results of microbial differences at the genus level in the DM and non-DM groups. **(D)** Heatmap of correlation analysis between the relative abundance of the top 10 microbiota at the genus level and the laboratory findings.

The statistical results of LEfSe included two parts, namely, a histogram of the LDA value distribution and a cladogram. The histogram of LDA values (Figure 3A) showed that there were 39 differentially abundant taxa at different taxonomic levels, of which 24 were from the DM group and 15 were from the non-DM group. The genus *Klebsiella* had the largest LDA score. The cladogram (Figure 3B) showed that the relative abundance of genus *Klebsiella* was greater in DM patients, when compared with non-DM patients.

Taxa relative abundance in the DM and non-DM groups for the discriminating genera is shown in Figure 3C. There were 17 discriminating genera between the two groups at the genus level, among which *Klebsiella* was the most discriminating taxa.

### Correlation analysis between pus microbiome composition and laboratory findings

To determine the functional relationships of microbiome composition and laboratory findings, we performed correlation analysis between the relative abundance of the top 10 microbiota at the genus level and using laboratory findings (Figure 3D). The results showed that the relative abundance of *Klebsiella* was positively correlated with blood glucose and CRP levels, which suggested that the relative abundance of *Klebsiella* was related to a high glucose environment and severe inflammatory reaction. The relative abundance of *Acidovorax* and *Faecalibacterium* was negatively correlated with CRP levels, but there was no correlation with blood glucose levels.

### Functional prediction using BugBase and PICRUSt2

As shown by the potential predictions of phenotypic functions in the bacterial community in PLA, nine potential microbial phenotypes were detected. We observed a significantly higher representation of those containing mobile elements bacteria, facultatively anaerobic bacteria, potential pathogenic bacteria, bacteria related to biofilm formation and stress-tolerant bacteria in the DM group, when compared with the non-DM group (Table 3).

**Table 3:** Bacterial phenotypes in the pus samples predicted by BugBase.

Phenotype	DM (*n* = 38) Relative abundance Mean (SD)	Non-DM (*n* = 24) Relative abundance Mean (SD)	*p*-value
Aerobic	0.04 (0.11)	0.15 (0.27)	0.087
Anaerobic	0.03 (0.05)	0.13 (0.29)	0.723
Facultatively anaerobic	0.92 (0.16)	0.68 (0.38)	**0.007**
Contain mobile elements	0.92 (0.16)	0.62 (0.42)	**0.006**
Form biofilms	0.92 (0.17)	0.62 (0.42)	**0.005**
Gram negative	0.97 (0.06)	0.86 (0.27)	0.164
Gram positive	0.03 (0.06)	0.14 (0.27)	0.164
Potentially pathogenic	0.92 (0.17)	0.61 (0.43)	**0.005**
Stress tolerant	0.92 (0.17)	0.61 (0.43)	**0.006**

SD, Standard Deviation. Bold value indicates *p* value <0.05.

**Figure 4:**
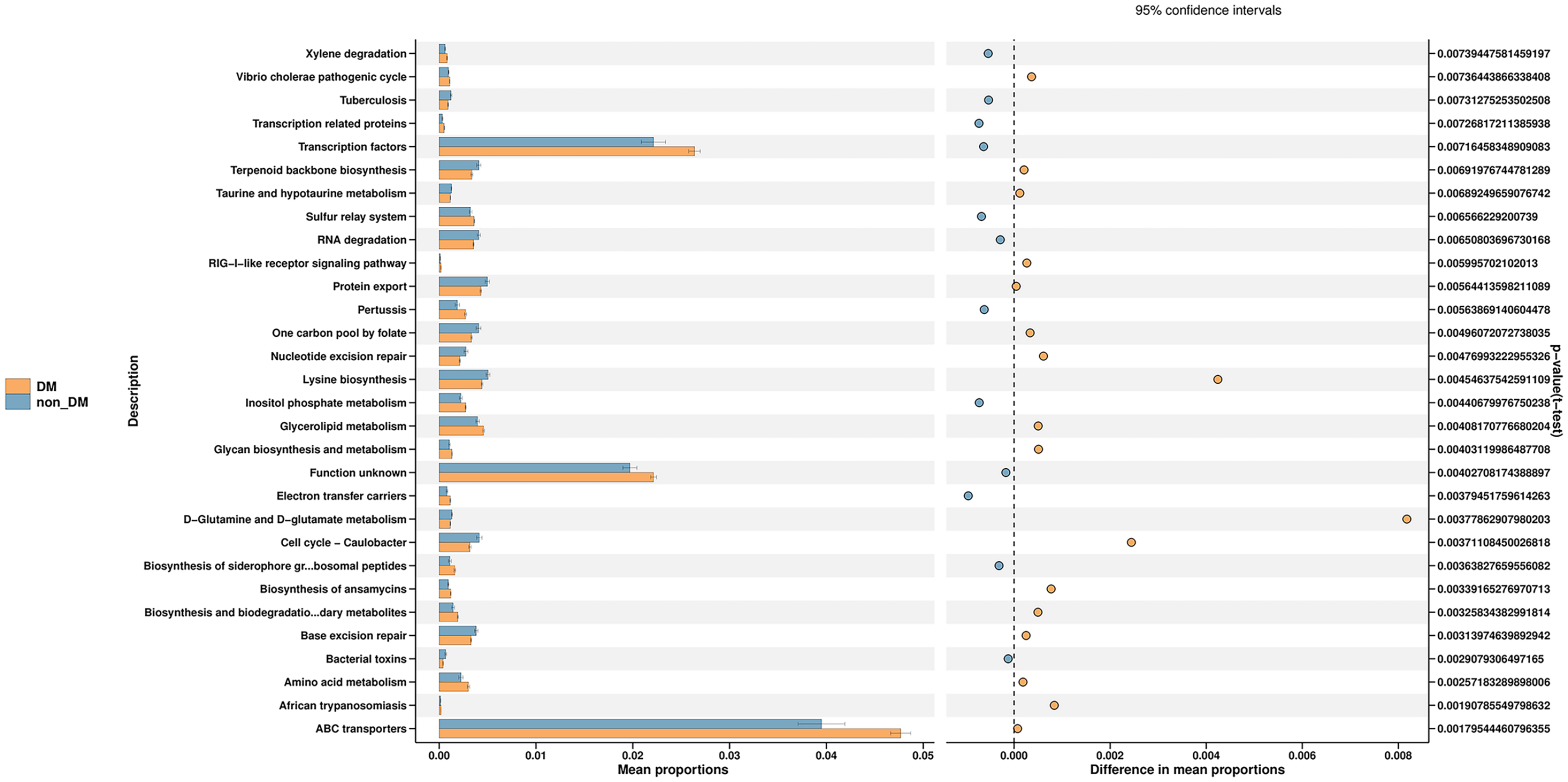
Prediction of microbial function in diabetic mellitus (DM) and non-DM groups based on the Kyoto Encyclopedia of Genes and Genomes database. The ABC transporters pathway was the most significant differential metabolic pathway in the top 30 pathways shown in the figure.

PICRUSt2 was used for functional profiling of the microbial community by using the KEGG database. Finally, we identified 38 KEGG pathways at level 3, which were significantly changed in percentage means between the two groups (*p* < 0.01). The top 30 pathway names sorted by *p*-values are included in Figure 4, with the most significant differential metabolic pathway involving ABC transporters.

### Pus metabolic differences between PLA patients with and without DM

**Figure 5:**
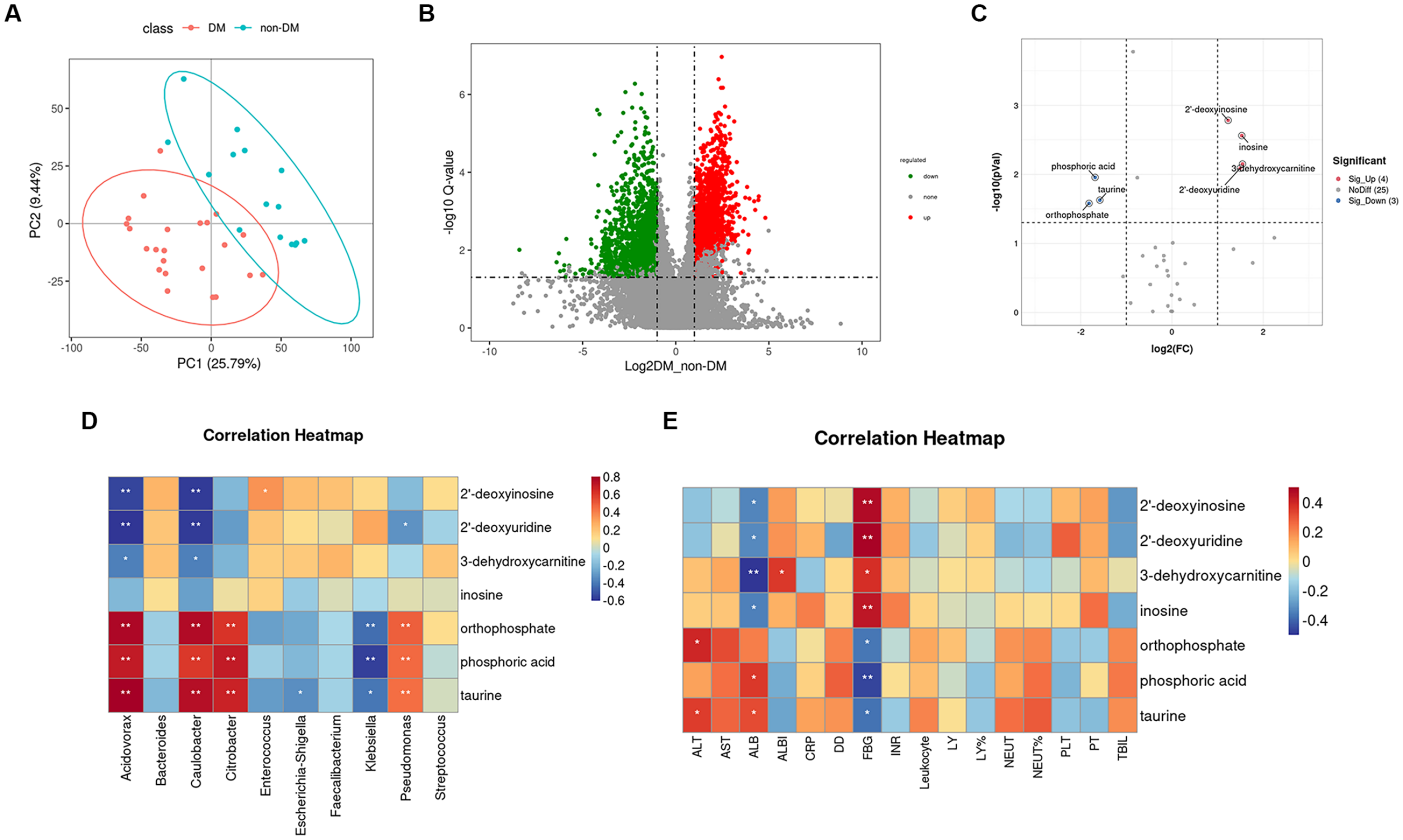
Partial least squares–discriminate analysis PLS-DA model, volcano plot, and heatmap of correlation analysis. **(A)** Score plot of the PLS-DA model in the diabetic mellitus (DM) and non-DM groups. **(B)** Volcano plot of differential metabolites between the two groups. The horizontal coordinate represents the fold change (log_2_FoldChange) of metabolites in different groups, and the vertical coordinate represents the significance level of the difference (−log_10_ *p* value). Each point in the volcano plot represents a metabolite, in which the metabolites with significantly up-regulated expressions are represented by red dots, and those with significantly down-regulated expressions are represented by green dots. The dot size variable importance for the projection values are represented by dot size. **(C)** Volcano plot of differential metabolites in the ABC transporters pathway between the two groups. **(D)** Heatmap of correlation analysis between the top 10 relative abundance microbiota at the genus level and the seven differentially accumulated metabolites in the ABC transporters pathway. **(E)** Heatmap of correlation analysis between the laboratory findings and the seven differentially accumulated metabolites in the ABC transporters pathway.

To further identify metabolomic changes in PLA patients with DM, we performed a non-targeted metabolomics study of 38 pus samples (DM, *n* = 22; non-DM, *n* = 16). PLS-DA, which has good discrimination ability, was used to analyze the metabolic profiles based on class information (Yu et al., 2021). The PLS-DA model showed that there was separation between the two groups (Figure 5A).

A volcano plot (Figure 5B) was constructed to show trends in differentially abundant metabolites between the two groups. Using KEGG enrichment analysis (Supplementary Figure S1), seven main pathways (Cellular Processes, Drug Development, Environmental Information Processing, Genetic Information Processing, Human Diseases Metabolism and Organismal Systems) were identified, with the most abundant being Metabolism. In this pathway, we found that the differential metabolites enriched in the DM group were mainly involved in amino acid biosynthesis and metabolism, lipid metabolism, glucose metabolism and polycyclic aromatic hydrocarbon degradation.

In the environmental information processing pathway, we found the ABC transporters pathway showed differences between the two groups, which was consistent with the 16SrDNA prediction. Based on variable importance in the projection values >1 in the loading plot, FC ≥ 2 or FC ≤ 0.5, and *p* < 0.05 with KEGG annotations, seven differentially accumulated metabolites were identified in the ABC transporters pathway, of which four metabolites (2′-deoxyuridine, 2′-deoxyinosine, 3-dehydroxycarnitine, and inosine) were enriched and three metabolites (phosphoric acid, taurine, and orthophosphate) were depleted (Supplementary Table S3; Figure 5C).

### Correlation analysis between differential metabolites in the ABC transporters pathway and pus microbiota composition

To identify functional relationships of pus microbiota and differentially accumulated metabolites in the ABC transporters pathway, we performed correlation analysis based on Pearson’s correlation coefficients. The top 10 relative abundance microbiota at the genus level and the above seven differentially-accumulated metabolites in the ABC transporters pathway were included for analysis, which showed that the metabolites was correlated with pus microbiota. The heatmap of the correlation is shown in Figure 5D. The results showed that phosphoric acid, taurine, and orthophosphate were positively correlated with *Acidovorax*, *Caulobacter*, *Citrobacter,* and *Pseudomonas,* while they were negatively correlated with *Klebsiella*. The 2′-deoxyuridine, 2′-deoxyinosine, and 3-dehydroxycarnitine were negatively correlated with *Acidovorax* and *Caulobacter.*

### Correlation analysis between the differential metabolites in the ABC transporters pathway and laboratory findings

Figure 5E shows the correlation heatmap for the seven differential metabolites in the ABC transporters pathway and the laboratory findings. The results suggested that all these metabolites were closely related to fasting blood glucose (FBG). The 2′-deoxyuridine, 2′-deoxyinosine, 3-dehydroxycarnitine and inosine were positively related to FBG, while phosphoric acid, taurine and orthophosphate were negatively correlated with FBG. These metabolites were also related to albumin (ALB), alanine aminotransferase (ALT) and albumin-bilirubin (ALBI), which are closely correlated with liver function.

## Discussion

The current study showed that PLA patients with DM had a higher CRP level, a higher EMI incidence and longer LOS, which is consistent with previous studies (Foo et al., 2010; Tian et al., 2012). Diabetes-induced functional defects in neutrophil responses to pathogenic microorganism, including impairments in phagocytosis, bacterial killing, neutrophil migration, cytokine production, apoptosis and neutrophil extracellular trap (NET) formation may contribute to the incidence of EMI in PLA patients, when combined with DM (Hodgson et al., 2015; Tay et al., 2018; Njeim et al., 2020). However, Lee et al. (2018) reported that type 2 DM did not overtly affect neutrophil intra- and extra-cellular killing of hypervirulent *Klebsiella pneumoniae*. Thus, the mechanism of severity of PLA patients with diabetes still needs to be fully determined.

Host immune defenses and pathogenic microorganisms affect the prognosis and the development of PLA. Identifying the differential microbiome composition differences between PLA patients with and without DM is therefore helpful in identifying potential causes. To the best of our knowledge, the current study is the first to use 16S rDNA sequencing to identify differences of microbiota composition between PLA patients with and without DM. Although the alpha diversity did not distinguish between the two groups, the results showed that the Simpson index linked with species richness and the Pielou-e index linked with species evenness were negatively correlated with the HbA1c levels, suggesting that a high glucose level may inhibit microbial diversity in the pus cavity. The influence of DM on intestinal flora abundance is still controversial. Some studies reported lower alpha diversity indices (Shannon and Chao) of gut microbiota in diabetic patients, when compared with healthy controls (Nuli et al., 2019; Zhao et al., 2019), while other studies reported increased microbial richness of gut microbiota in diabetic patients (Bai et al., 2022). The effect of DM on intestinal flora and microorganisms in pus may also differ.

*Klebsiella*, whose relative abundance was higher in the DM group, was the most discriminating genus between the two groups and the relative abundance of *Klebsiella* was positively correlated with blood glucose and CRP levels. Therefore, we speculate that the severe inflammation in the DM group was partially due to the higher relative abundance of *Klebsiella*. Previous studies reported that *Klebsiella* pneumonia liver abscess tended to have a higher incidence of EMI, especially in DM patients (Yoon et al., 2014; Xu et al., 2020). Lee et al. (2018) postulated that the mechanism may be that Klebsiella pneumonia might activate platelet activity especially in DM patients with poor glycemic control and form a septic thrombosis, which can lead to seeding of microorganisms with metastatic infections.

Based on the prediction of BugBase, we observed a significantly higher representation of those containing mobile elements bacteria, facultatively anaerobic bacteria, potential pathogenic bacteria, bacteria related to biofilm formation and stress-tolerant bacteria in the DM group, when compared with the non-DM group. Biofilm formation plays an important role in antibiotic resistance and escape of microbes from the body’s immune surveillance system (Rather et al., 2021). Stress tolerance is a characteristic that improves resistance toward adverse environmental abiotic and biotic stress factors (Vassilev et al., 2012). Differences in these bacterial phenotypes may be one of the reasons that PLA patients with DM have more serious clinical manifestations.

Another noteworthy finding was that the ABC transporters pathway was the most significant differential metabolic pathway in KEGG pathways predicted by PICRUSt2. The ABC transporters superfamily is among the largest and most broadly expressed protein superfamily, is found in all living organisms and is involved in almost every cellular, biological and physiological system (Mercado-Lubo and McCormick, 2010). ABC transporters in the accessory genomes of bacterial pathogens significantly influence both virulence and antimicrobial resistance (Theodoulou and Kerr, 2015; Farzand et al., 2021). There was a large number of differential metabolites between the two groups, of which seven belonged to the ABC transporters pathway. All of these seven metabolites were closely related to FBG. Some of them were correlated with liver function and the relative abundance of microbiota, suggesting that these metabolites may contribute to the more severe clinical manifestations in the DM group. Unfortunately, there are few reports on the functions of these metabolites, which need further study in the future.

There were several limitations to this study. First, the current study is an exploratory study, so we did not perform a power analysis to estimate the sample size. Second, our study only detected the metabolic situation in pus samples without untargeted metabolomics analysis on serum samples, which can better reflect the metabolic situation of the body. In addition, it was difficult to determine whether the differential metabolites between the two groups detected in pus samples came from the host or microorganisms in pus. It is therefore necessary to collect both pus samples and blood samples in future research.

In conclusion, PLA patients with DM had more severe clinical manifestations than PLA patients without DM. The relative abundance of *Klebsiella* in the pus cavity of PLA patients with DM was higher than in PLA patients without DM and was accompanied by changes of various metabolites and metabolic pathways, which may be associated with the more severe clinical manifestations.

## Data availability statement

The datasets presented in this study can be found in online repositories. The names of the repository/repositories and accession number(s) can be found in the article/Supplementary material.

## Ethics statement

The studies involving human participants were reviewed and approved by The Ethics Committee of Shengjing Hospital of China Medical University. The patients/participants provided their written informed consent to participate in this study.

## Author contributions

ZC and YG designed the study and analyzed and interpreted the data. YG and HW collected data. YG, ZL, and ZC wrote the manuscript. All authors contributed to the article and approved the submitted version.

## Funding

The present study was supported by the National Natural Science Foundation of China (81901856 and 82272097) and the 345 Talent Project in Shengjing Hospital of China Medical University.

## Conflict of interest

The authors declare that the research was conducted in the absence of any commercial or financial relationships that could be construed as a potential conflict of interest.

## Publisher’s note

All claims expressed in this article are solely those of the authors and do not necessarily represent those of their affiliated organizations, or those of the publisher, the editors and the reviewers. Any product that may be evaluated in this article, or claim that may be made by its manufacturer, is not guaranteed or endorsed by the publisher.
