# Supplementary figures and images for: Comprehensive analysis of the microbiome and metabolome in pus from pyogenic liver abscess patients with and without diabetes mellitus
Source: Front Microbiol. 2023 Jun 23;14:1211835. doi: 10.3389/fmicb.2023.1211835 (PMC10328747; doi:10.3389/fmicb.2023.1211835)

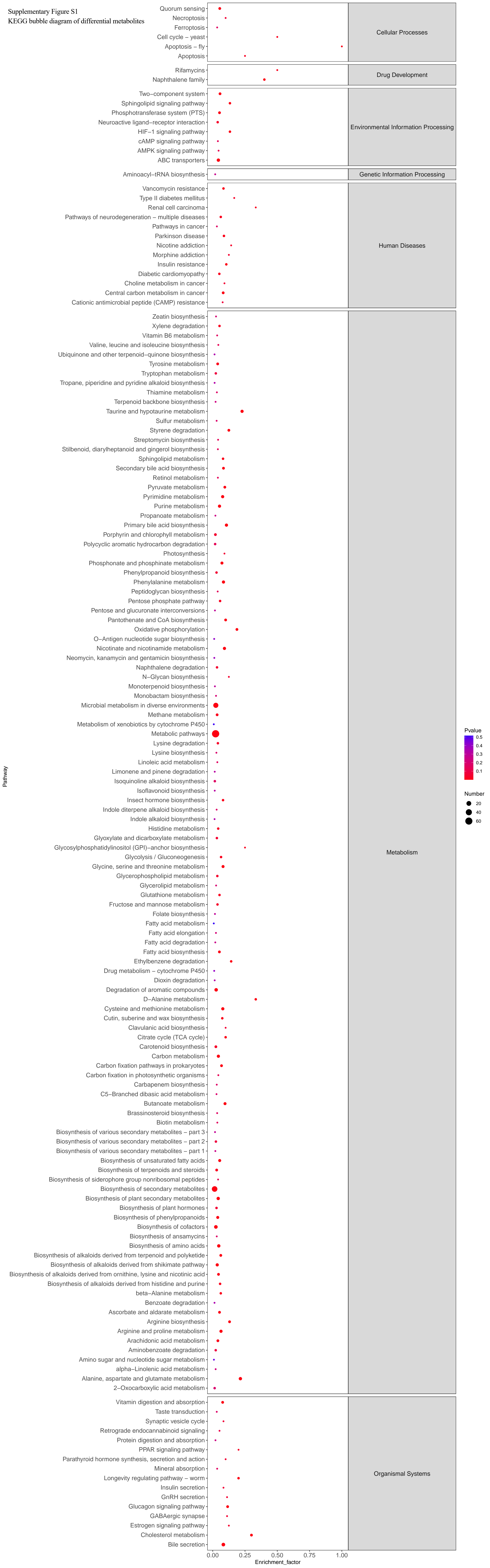

Supplement: Supplementary file 5 [file Image_1.pdf]
